# Toxoplasmosis: a rare cause of IRIS in HIV infected patients. Case series

**DOI:** 10.1186/1471-2334-13-S1-O7

**Published:** 2013-12-16

**Authors:** Ruxandra Moroti, Daniela Munteanu, Mihaela Rădulescu, Adriana Hristea, Iulia Niculescu, Raluca Mihăilescu, Roxana Petre, Raluca Hrişcă, Raluca Jipa, Ana Maria Petrescu, Maria Nica, Mihai Lazăr, Anca-Ruxandra Negru, Irina Lăpădat, Angelica Teniță, Victoria Aramă

**Affiliations:** 1 National Institute for Infectious Diseases “Prof. Dr. Matei Balş”, Bucharest, Romania; 2 Carol Davila University of Medicine and Pharmacy, Bucharest, Romania; 3 Central Universitary Emergency Military Hospital Dr Carol Davila, Bucharest, Romania; 4 Clinical Hospital of Infectious and Tropical Diseases “Dr. Victor Babeş”, Bucharest, Romania

## Background

Cerebral toxoplasmosis is one of the main 3 intracerebral opportunistic infections in HIV positive patients, along with cryptococcosis and tuberculosis. In comparison to these last 2 entities, toxoplasmosis does not provoke or very rarely provokes reconstitution syndromes.

## Methods

We analyzed a case series of 3 patients with cerebral toxoplasmosis admitted in the Adults III Department of the National Institute for Infectious Diseases “Prof. Dr. Matei Balş” in 2012-2013.

## Results

Three patients, one male and 2 women, aged 55 years old, respectively 41 and 42 year-old, all 3 diagnosed concomitantly with HIV infection (as very late presenters) and cerebral toxoplasmosis, with a CD4 count of 6, 6 and 7/cmm respectively, viral loads (VL) of 254,000, 57,000 and 156,000 copies/mL respectively, and CSF viral load below the plasmatic VL in all 3 cases. We recorded minimal abnormalities of CSF analysis regarding the number of cells and biochemical exams; all had positive PCR for *Toxoplasma gondii* in the CSF and positive serology (IgG). All 3 had intracerebral lesions (abscesses) and all were biopsied at the neurosurgery department for diagnostic purpose before knowing their HIV-positive status. They received high doses of oral trimethoprim/sulfamethoxazole (T/S) for toxoplasmosis and antiretroviral therapy in the first 2 weeks after the diagnosis. They repeated cerebral imagery (MRI) after 3 weeks of T/S and had no regression of the size of lesions (although with the decreasing of perilesional edema) and new lesions, in two cases without having corresponding symptoms; in all 3 cases the CD4 count increased in the first month more than 100%. The search for another cause for the augmentation of their brain lesions was negative. Maintaining the same medication, the next imagery exams showed improvement in 2 out of 3 cases, in which the outcome was favorable with almost complete neurological recovery. In the remaining case the evolution was unfavorable (death).

## Conclusions

In our 3 cases we presumed a paradoxical toxoplasmosis IRIS, with little or no clinical deterioration strictly linked with imagery exams depreciation in 2 out of 3 cases but with a fatal evolution in one case. Even rarely reported, the toxoplasmosis IRIS could be taken into account in some situations.

